# Supplementary material for: In-Hospital Mortality Among Patients Undergoing Percutaneous Pericardiocentesis for Pericardial Effusion with and Without Malignancy
Source: Curr Oncol. 2025 Sep 15;32(9):514. doi: 10.3390/curroncol32090514 (PMC12468728; doi:10.3390/curroncol32090514)
Supplement: Supplementary file 1 [file curroncol-32-00514-s001.zip › curroncol-3825341-supplementary.pdf]

Table S1. List of the ICD-10-CM codes used.

Diagnosis	ICD-10 Code
Pericardiocentesis*	0W9D3, 0W9D4, 02BN3, 02BN4, 02NN3, 02NN4, 02FN3, 02FN4
Percutaneous coronary intervention*	027034, 027035, 027036, 027037, 027044, 027045, 027046, 027047, 027134, 027135, 027136, 027137, 027144, 027145, 027146, 027147, 027234, 027235, 027236, 027237, 027244, 027245, 027246, 027247, 027334, 027335, 027336, 027337, 027344, 027345, 027346, 027347, 02703D, 02703E, 02703F, 02703G, 02704D, 02704E, 02704F, 02704G, 02713D, 02713E, 02713F, 02713G, 02714D, 02714E, 02714F, 02714G, 02723D, 02723E, 02723F, 02723G, 02724D, 02724E, 02724F, 02724G, 02733D, 02733E, 02733F, 02733G, 02734D, 02734E, 02734F, 02734G, 02703Z, 02704Z, 02713Z, 02714Z, 02723Z, 02724Z, 02733Z, 02734Z
Cardiac surgery*	02100, 02110, 02120, 02130, 02RF07, 02RF08, 02RF0K, 02RF0J, 02RF47, 02RF48, 02RF4J, 02RF4K, 02QF0Z, 02QF4Z, 02UF07, 02UF08, 02UF0J, 02UF0K, 02UF47, 02UF48, 02UF4J, 02UF4K, 02RG07, 02RG08, 02RG0J, 02RG0K, 02RG47, 02RG48, 02RG4J, 02RG4K, 02QG0Z, 02QG4Z, 02UG07, 02UG08, 02UG0J, 02UG0K, 02UG47, 02UG48, 02UG4J, 02UG4K, 02RJ07, 02RJ08, 02RJ0J, 02RJ0K, 02RJ47, 02RJ48, 02RJ4J, 02RJ4K, 02QJ0Z, 02QJ4Z, 02UJ07, 02UJ08, 02UJ0J, 02UJ0K, 02UJ47, 02UJ48, 02UJ4J, 02UJ4K, 02RH07, 02RH08, 02RH0J, 02RH0K, 02RH47, 02RH48, 02RH4J, 02RH4K, 02QH0Z, 02QH4Z, 02UH07, 02UH08, 02UH0J, 02UH0K, 02UH47, 02UH48, 02UH4J, 02UH4K
Endomyocardial biopsy*	B216ZCZ
Cardiac catheter ablation*	02583ZZ, 02584ZZ
Pacemaker placement*	0JH604Z, 0JH605Z, 0JH606Z, 0JH634Z, 0JH635Z, 0JH636Z, 0JH804Z, 0JH805Z, 0JH806Z, 0JH834Z, 0JH835Z, 0JH836Z, 02H40JZ, 02H43JZ, 02H44JZ, 02H40NZ, 02H43NZ, 02H44NZ, 02H60JZ, 02H63JZ, 02H64JZ, 02H60NZ, 02H63NZ, 02H64NZ, 02H70JZ, 02H73JZ, 02H74JZ, 02H70NZ, 02H73NZ, 02H74NZ, 02HK0JZ, 02HK3JZ, 02HK4JZ, 02HK0NZ, 02HK3NZ, 02HK4NZ, 02HL0JZ, 02HL3JZ, 02HL4JZ, 02HL0NZ, 02HL3NZ, 02HL4NZ
Implantable cardioverter-defibrillator*	02HK0KZ, 02HK3KZ, 02HK4KZ, 0WHC0GZ, 0WHC3GZ, 0WHC4GZ, 02H40KZ, 02H43KZ, 02H44KZ, 02H60KZ, 02H63KZ, 02H64KZ, 02H70KZ, 02H73KZ, 02H74KZ, 02HL0KZ, 02HL3KZ, 02HL4KZ, 02HN0KZ, 02HN3KZ, 02HN4KZ, 0JH608Z, 0JH60FZ, 0JH638Z, 0JH63FZ, 02H44KZ, 02H64KZ, 02H74KZ, 02HL4KZ, 02HK4KZ
Cardiac resynchronization therapy*	0JH607Z, 0JH609Z, 0JH637Z, 0JH639Z, 0JH807Z, 0JH809Z, 0JH837Z, 0JH839Z
Smoking	F17, T65, Z72.0, O99.33, Z87.891
Hypertension	I10
Diabetes mellitus	E08, E10, E11, E13
Hyperlipidemia	E78
Obesity	E66
Congestive heart failure	I09.81, I11.0, I13.0, I31.2, I50
Chronic ischemic heart disease	I25
Atrial fibrillation	I48.0, I48.1, I48.2, I48.91
Chronic obstructive pulmonary disease	J41, J42, J43, J44
Pulmonary hypertension	I27.0, I27.2
Chronic kidney disease	N18
End-stage renal disease	N18.6
Liver cirrhosis	K70.2, K70.3, K71.7, K74, K76.1, P78.81, E83.110
Dementia	F01, F02, F03, G30, G31
Anemia	D50, D51, D52, D53, D63, D64
Thrombocytopenia	D69.4, D69.5, D69.6
Malnutrition	E43, E44, E46
Coagulopathy	D65, D66, D67, D68, D69

Hypothyroidism	E03
Acute pericarditis	I30
Infective endocarditis	I33, A32.82, A39.51, A52.03, B33.21, B37.6
History of irradiation	Z92.3
Systemic lupus erythematosus	M32, L93
Rheumatoid arthritis	M05, M06
Systemic sclerosis	M34
Inflammatory myopathy	M33
Sjogren's disease	M35.0
Sarcoidosis	D86
Oropharyngeal cancer	C00, C01, C02, C03, C04, C05, C06, C07, C08, C09, C10, C11, C12, C13, C14
Esophageal cancer	C15
Gastric cancer	C16
Colorectal cancer	C18, C19, C20
Anal cancer	C21
Hepatobiliary cancer	C22, C23, C24
Pancreatic cancer	C25
Lung cancer	C34
Skin cancer	C43, C44
Breast cancer	C50
Cervical cancer	C53
Uterine cancer	C54, C55
Ovarian cancer	C56
Prostate cancer	C61
Renal cancer	C64, C65
Bladder cancer	C67
Central nervous system cancer	C69, C70, C71, C72
Thyroid cancer	C73
Hodgkin lymphoma	C81
Non-Hodgkin lymphoma	C82, C83, C84, C85, C86, C88
Multiple myeloma	C90
Leukemia	C91, C92, C93, C94, C95
Other cancers	C17, C26, C30, C31, C32, C33, C37, C38, C39, C40, C41, C45, C46, C47, C48, C49, C51, C52, C57, C58, C60, C62, C63, C66, C68, C74, C75, C76, C77, C78, C79, C80, C7A, C7B, C96
Metastasis	C77, C78, C79, C80.0, C80.2, R18.0, C7B
Pericardial tamponade	I31.4
Surgical pericardial drainage*	0W9D0, 02BN0, 02NN0, 02FN0
Mechanical ventilation*	5A1935Z, 5A1945Z, 5A1955Z
Need of vasopressor support	3E043XZ, 3E033XZ

*ICD-10-PCS codes

Abbreviations: ICD-10-CM, International Classification of Diseases, Tenth Revision, Clinical Modification; ICD-10-PCS, International Classification of Diseases, Tenth Revision, Procedure Coding System
